# 

**Published:** 1900-09-22

**Authors:** 


					the hospital. "The standard of highest parity?Lancet, September 22sd, 1900.
CADBURY'S M* U *?4 CADBURY'S
COOOA  )?> COCOA
NO DRUGS OR ALKALIES USED.
SSp^^lASCotv Western Infirm ah*
No. 73p. Vol. XXVIII. Saturday,
September 22, 1900.
"Bnbsor^tion Terms,] pRICE TWOPENCE.

				

